# Barium Titanate-Based Glass–Ceramics Crystallized from Multicomponent Oxide Glasses: Phase Composition and Microstructure

**DOI:** 10.3390/ma18163783

**Published:** 2025-08-12

**Authors:** Ruzha Harizanova, Wolfgang Wisniewski, Dragomir M. Tatchev, Georgi Avdeev, Svetlozar Nedev, Christian Rüssel

**Affiliations:** 1 Physics Department, University of Chemical Technology and Metallurgy, 8 Kl. Ohridski Blvd., 1756 Sofia, Bulgaria; 2 Institute of Materials Science and Engineering, Chemnitz University of Technology, Erfenschlager Str. 73, 09125 Chemnitz, Germany; 3 Otto Schott Institute for Materials Research, University of Jena, Fraunhoferstr. 6, 07743 Jena, Germany; ccr@uni-jena.de; 4 Institute of Physical Chemistry, Bulgarian Academy of Sciences, Block 11, Acad. G. Bonchev Str., 1113 Sofia, Bulgaria; dtachev@ipc.bas.bg (D.M.T.); g_avdeev@ipc.bas.bg (G.A.)

**Keywords:** barium titanate, zirconium oxide, barium fresnoite, phase separation, glass–ceramics, X-ray diffraction, scanning electron microscopy, X-ray tomography, dielectric properties, relaxors

## Abstract

The interest in synthesizing new dielectric materials is caused by their potential application in various electronic and sensor devices as well as in a large variety of electronic components. The present work reports the synthesis of glasses in the Na_2_O/Al_2_O_3_/BaO/ZrO_2_/TiO_2_/B_2_O_3_/SiO_2_ system prepared by melt-quenching. These glasses were then crystallized to glass–ceramics by a controlled thermal treatment. X-ray diffraction experiments reveal the precipitation of Ba_2_TiSi_2_O_8_ (fresnoite) and BaTiO_3_, which probably forms a BaZr_x_Ti_1−x_O_3_ solid solution. The microstructure is studied by scanning electron microscopy and shows the presence of mulberry-shaped, crystallized structures with a densely-branching morphology. Microcomputed X-ray tomography is used to gather information on the volume fraction and average size of the crystallized volume as an effect of the applied temperature–time schedule. Longer annealing times lead to a higher volume fraction and increasing average size of the crystallization structures obtained. The dielectric properties analyzed by impedance spectroscopy are insulating and show relatively high dielectric constants ≥ 100 and moderate loss tangent values at 10 kHz.

## 1. Introduction

Controlled crystallization has often been used to prepare dielectric materials with high dielectric constants and low losses from complex oxide glasses [1,2,3,4,5,6,7]. The main approach is to choose a well-investigated base glass and add the constituents required for synthesizing the desired dielectric phase. Applying this concept allows us to investigate the effect of the added components on the glass structure and the crystallization behavior of the prepared materials [1]. The glass is usually prepared by melt quenching, ground to a powder, compacted, and subjected to sintering by applying appropriate time–temperature schedules. This enables the preparation of glass–ceramic materials with controllable dielectric properties [4,5,6,7]. Another approach which does not require the outlined two-stage preparation is to choose a chemical composition which, after melting and quenching, will phase separate during the thermal treatment. Here, the initial compositions can be selected so that one subsequently separated phase mainly contains the elements of the pre-desired dielectric phase whereas the other is enriched in all other glass components [1,3]. One well-investigated dielectric phase successfully crystallized from multicomponent oxide glasses is BaTiO_3_ [2], which is of interest due to its potential for application in various devices in the fields of electronics, sensors, and energy storage [2,8,9,10,11,12].

Applications for BaTiO_3_-based materials depend on the four polymorphic modifications of BaTiO_3_ which, to a variable extent, exhibit such properties as piezoelectricity, pyroelectricity, and ferroelectricity. Above its Curie temperature (T_Curie_) of 120–130 °C, BaTiO_3_ occurs in its cubic modification [2,4,5,6,7,8,9], where Ba^2+^ ions occupy the corners of the unit cell, Ti^4+^ ions are centered in the cross-section of the space diagonals, and O^2−^ ions are centered at the cube faces. Cubic BaTiO_3_ is an isotropic dielectric with paraelectric properties. Below T_Curie_, BaTiO_3_ shows a tetragonal structure where the Ba^2+^ and O^2−^ ions are, respectively, located at the corners and the face centers, while the Ti^4+^ ions are shifted towards the (001)- or (00$\vec{1}$)—planes. The latter induces a dipolar moment parallel to the crystallographic c-axis and a spontaneous polarization, i.e., it leads to ferroelectric and piezoelectric properties. Further decreasing the temperature leads to an orthorhombic modification at approximately 0 °C and then to a rhombohedral phase at –90 °C [2,8,9]. The dielectric properties of BaTiO_3_ have been modified and controlled by doping with other components, such as SrO and ZrO_2_ [13,14,15,16,17,18,19,20,21,22,23]. For example, the phase transition temperatures can be widely affected by substituting Ba^2+^ with other di- or even trivalent cations or, alternatively, Ti^4+^ can be replaced by Zr^4+^ [22,23] or other tri-, tetra-, and/or pentavalent cations. One main problem in the performance of such ceramics is that their microstructure is inhomogeneous, and unreacted raw materials, such as BaCO_3_, can occur in the final product. Producing these phases in glass–ceramics via controlled crystallization from glasses is frequently proposed as a solution of this problem [2,12,13,19] as it enables researchers to obtain materials with a high purity, narrow and homogeneous particle size distributions, and a controllable degree of crystallinity [2,12].

Prospective applications require different properties; thus, tailoring the phase composition and microstructure is important [3]. Glasses from the compositional series 20.1 Na_2_O∙(23.1-y) BaO∙y ZrO_2_∙23 TiO_2_∙9.8 B_2_O_3_∙21 SiO_2_∙3 Al_2_O_3_ with y = 0, 0.5, 1, 2, and 3 mol% have been prepared using the well-studied system Na_2_O/B_2_O_3_/SiO_2_/Al_2_O_3_ and adding large concentrations of BaO and TiO_2_; some of the BaO was substituted by equimolar concentrations of ZrO_2_, as reported in Ref. [24]. The proposed compositions enable the preparation of glass–ceramic materials containing crystalline BaTiO_3_ and/or BaTi_1−x_Zr_x_O_3_.

The work presented here features glass–ceramics obtained from the glasses in the compositional series noted above [24] via controlled crystallization. The glass–ceramics are investigated by X-ray diffraction (XRD) and scanning electron microscopy (SEM), including energy-dispersive X-ray spectroscopy (EDXS) as well as microcomputed X-ray tomography (XTM). Impedance spectroscopy is used to investigate the dielectric properties of the prepared glass–ceramics.

## 2. Materials and Methods

### 2.1. Materials

The glasses with the composition 20.1 Na_2_O∙(23.1-y) BaO∙y ZrO_2_∙23 TiO_2_∙9.8 B_2_O_3_∙21 SiO_2_∙3 Al_2_O_3_ with y = 0, 0.5, 1, 2, and 3 mol% were prepared by conventional melt quenching from the reagent-grade raw materials Na_2_CO_3_, ZrO_2_, SiO_2_, BaCO_3_, B(OH)_3_, Al(OH)_3_, and TiO_2_. Batches of 60 g were homogenized and melted in Pt crucibles using a MoSi_2_ furnace and a temperature of 1400 °C supplied for 1 h in air. The melts were quenched on a Cu block without pressing, transferred into a pre-heated graphite mold, and finally transferred to a muffle furnace, where they were held at 450 °C for 10 min. The furnace was then switched off, allowing the samples to cool to room temperature with a rate of approximately 2 K/min. The samples are denoted for their ZrO_2_ content, i.e., y = 0, 0.5, 1, 2, and 3 mol% as 0Zr, 05Zr, 1Zr, 2Zr, and 3Zr, respectively.

Pieces of ca. 10 × 10 × 5 mm^3^ were cut from the glass blocks and thermally treated by applying appropriate time–temperature schedules, according to the data listed in Table 1.

### 2.2. Methods

X-ray diffraction in the Bragg–Brentano (θ-2θ) setup (XRD, X-ray diffractometer Empyrean, Malvern Panalytical, Almelo, Netherlands) was performed using Cu-Kα radiation (λ = 1.5406 Å) and a Ni filter in the 2θ range from 10 to 60°. Rietveld analysis was carried out for the quantification of the crystallite sizes of the phases detected and the determination of the fractions of each detected phase. For Rietveld refinements, HighScore Plus 4.0 software was used.

SEM analyses were carried out using a Jeol JSM 7001F SEM (JEOL GmbH, Freising, Germany) equipped with an EDAX Trident analyzing system. EDXS was performed without a standard using an acceleration voltage of 15 kV. The samples for SEM imaging were cross-fractured, embedded in araldite epoxy resin and polished to optical quality before applying a thin carbon layer to avoid charge up. The average particle size and volume fraction of the crystalline phase were evaluated without additional preparation by XTM (X-ray tomograph SkyScan 1272, Brucker microCT, Kontich, Belgium) applying a voltage of 100 kV and a current of 100 µA to the X-ray tube. The 3D voxel size was 0.8 µm^3^ and the scanning time about 6 h. The reconstruction of the images was performed using the software NRecon v2.2.0.6 including corrections for circular artifacts and changes in the radiation spectrum due to preferential absorption of low energy X-ray radiation. The results were visualized using DataViewer and CtVox from the CtAn package (Brucker). The data processing and evaluation were performed according to the procedure described in [25].

Electrical measurements were performed using impedance spectroscopy. Cylindrical samples (diameter of 7–10 mm, thickness of ca. 2–3 mm) were polished plane-parallel, coated with Ag paste on the opposing flat surfaces, and connected to Pt-plate electrodes with a diameter of 12 mm. They were then dried at 300 °C for 0.5 h, mounted into the sample holder, and connected to the impedance analyzer (Zahner IM6, Zahner GmbH, Kronach, Germany). Two contact point measurements and an ac voltage with an amplitude of 500 mV were used. The impedance modulus and the phase angle were measured as a function of the frequency (10 Hz–5x10^5^ Hz) and temperature (from room temperature to 200 °C), with an error ≤ 2%. The capacitances derived from the equivalent circuit, assuming RC (a resistor and a capacitor) in parallel, were determined at different frequencies and enabled the calculation of the corresponding dielectric complex permittivity from the sample geometry.

## 3. Results

Details of the glass preparation and their structural, thermophysical, mechanical, and optical properties were reported in Ref. [24]. The reported glass transition temperature (T_g_) and crystallization peak maximum temperatures (T_c1_, T_c2_) listed in Table 1 served as the basis for choosing appropriate time–temperature schedules for preparing the glass–ceramics. The heat treatment temperatures of 590 or 680 °C were chosen because of their proximity to the two crystallization peak maxima determined by DSC, and the glasses were heat-treated for various periods of time. The obtained samples were milky opaque with a white coloration, while the respective glasses were transparent to visible light [24].

### 3.1. XRD

**Figure 1:**
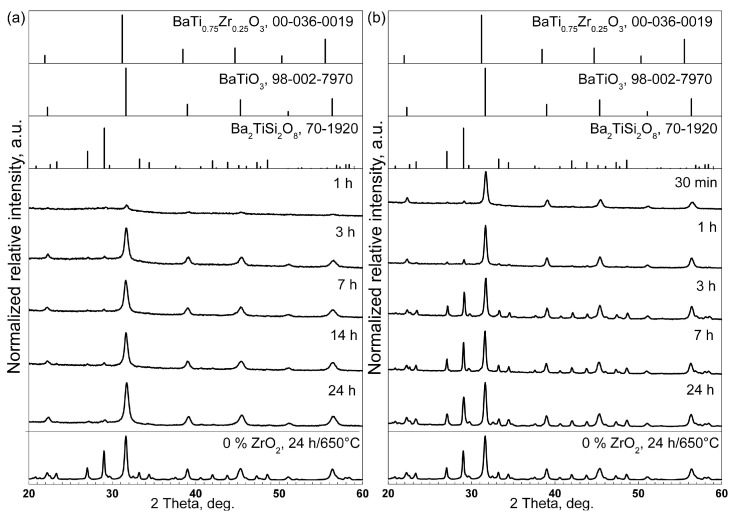
XRD patterns of 1Zr samples heat-treated at (**a**) 590 °C or (**b**) 680 °C for the stated periods of time. The XRD pattern of a sample with 0Zr heat-treated at 650 °C for 24 h [26] as well as the calculated patterns of the respectively stated phases are provided for reference.

Fine powders prepared from the annealed samples were analyzed using XRD and resulting patterns are presented in Figure 1. The XRD results show that all samples contain cubic BaTiO_3_ as the primary crystalline phase, and the most intense peak of fresnoite is also discernible in the patterns of all samples crystallized for at least 3 h. The peak positions match those of BaTiO_3_ rather than those of the presented reference pattern of the BaTi_0.75_Zr_0.25_O_3_ solid solution containing 25 at% Zr. As the maximum average Zr content in the prepared melts is much lower, it would likely need a large concentration of Zr into these crystals to enable a reliable statement about the formation of a BaZr_x_Ti_1−x_O_3_ solid solution based on XRD. Nevertheless, the literature [22,23] clearly states that Zr is included into the BaTiO_3_ lattice; hence, these crystals will subsequently be referred to as “BaZr_x_Ti_1−x_O_3_”. Longer annealing times of up to 24 h at 590 °C do not result in the occurrence of further diffraction peaks. Crystallizing at 680 °C for time periods ≥ 1 h leads to much stronger peaks matching the positions of fresnoite. Very small diffraction domains have been reported to affect the EBSD pattern formation of BaTiO_3_ in related microstructures [26]. Hence, the relatively wide crystallization peaks of BaZr_x_Ti_1−x_O_3_ likely result from XRD line broadening due to precipitated perovskite phase. However, the peak broadening for this crystalline phase due to the occurrence of solid solutions with different chemical compositions should also be considered, bearing in mind the compositional series developed. It is noteworthy that the mulberry-like structures showed no preferred orientation of the formed BaTiO_3_ crystals [26].

**Figure 2:**
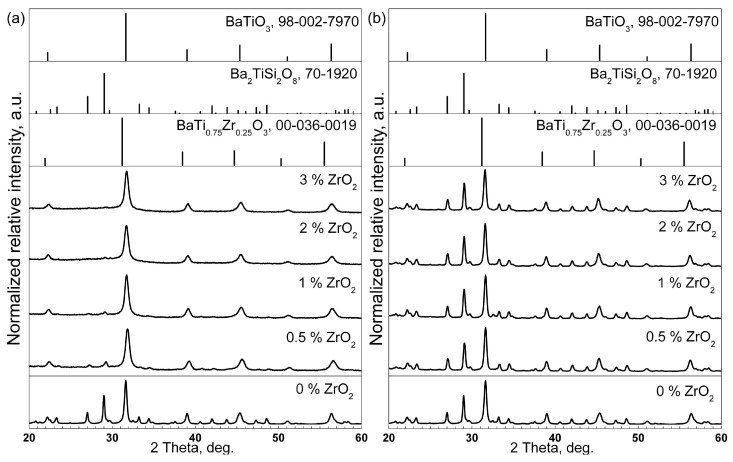
XRD patterns of 05Zr, 1Zr, 2Zr, and 3Zr samples heat-treated at (**a**) 590 °C and (**b**) 680 °C for 24 h. The XRD pattern of the 0Zr sample heat-treated at 650 °C for 24 h is given as a reference.

The XRD patterns of the obtained glass–ceramics with different thermal histories are presented in Figure 2. Samples heat-treated at 590 °C for 24 h (see Figure 2a) show that the quantity of fresnoite decreases with increasing ZrO_2_ concentrations. Actually, other crystalline phases are basically not indicated in the XRD patterns of the samples with different ZrO_2_ concentrations. In Figure 2b, XRD patterns are shown for an annealing temperature of 680 °C and 24 h. The XRD patterns for different ZrO_2_ concentrations show a similar shape.

### 3.2. SEM

The microstructure and local chemical composition of the prepared glass–ceramics was studied by SEM combined with EDXS.

**Figure 3:**
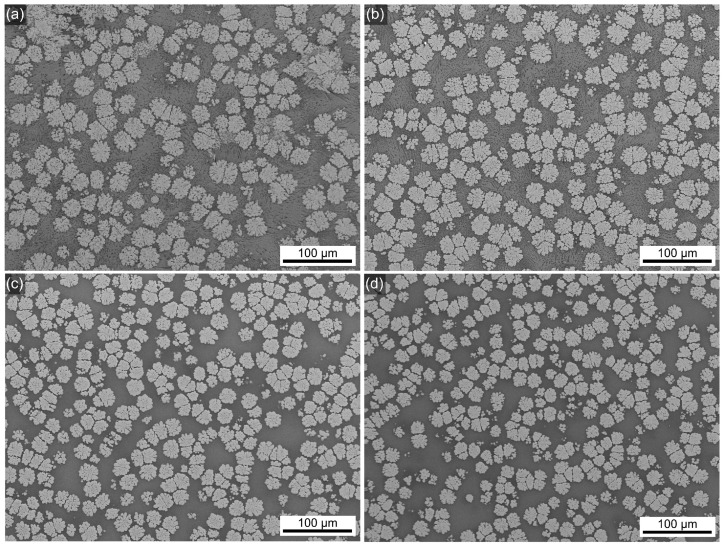
SEM micrographs of sample cross sections with (**a**) 0.5, (**b**) 1, (**c**) 2, or (**d**) 3 mol% ZrO_2_ heat-treated for 24 h at 590 °C.

**Figure 4:**
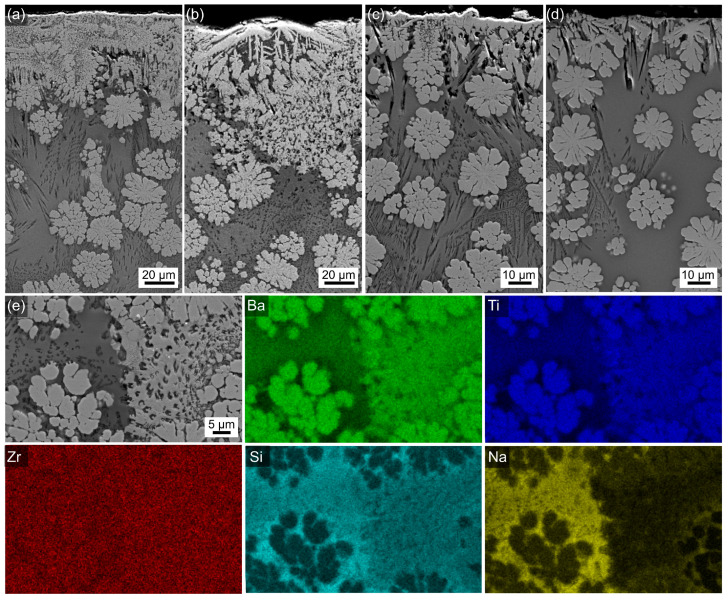
SEM micrographs presenting the surface crystallization layer in samples with (**a**) 0.5, (**b**) 1, (**c**) 2, or (**d**) 3 mol% ZrO_2_ heat-treated for 24 h at 590 °C. (**e**) An area of secondary crystallization in the bulk of the sample with 0.5 mol% ZrO_2_ as well as selected EDXS element maps of this area.

The bulk morphology of all glass–ceramic samples looks similar in the overviews presented in Figure 3, showing mulberry-shaped structures which are separated by a matrix of darker contrast. The morphology of these crystalline structures was also reported for the glass–ceramics prepared from the 0Zr composition [26] and discussed in Ref. [3]. Figure 4 shows all samples to have a layer of surface crystallization, decreasing in thickness with increasing ZrO_2_ content, as well as secondary crystallization in the matrix. The morphologies indicate the presence of two more crystalline phases, i.e., one of bright SEM contrast, equal to BaZr_x_Ti_1−x_O_3_, not showing the mulberry-shape and systematically occurring at the surface, and another of dark contrast forming very fine structures which result from dendritic crystal growth [3] throughout the bulk. The element maps in Figure 4 clearly show the secondary, non-mulberry-shaped phase of bright SEM contrast to contain more Ba, Ti, and Si than the mulberry-shaped structures, and EDXS spot measurements indicated this to be fresnoite. Na and Si are enriched in the matrix which can be assumed to represent the residual glass. The EDXS map of Zr shows little contrast, indicating a relatively homogeneous distribution in this sample containing only a small amount of Zr. However, comparable results were obtained for samples containing more Zr which supports the line broadening in Figure 1 to result from small diffraction domains rather than a second phase. The homogeneous distribution of Zr proves that significant accumulations of Zr into BaZr_x_Ti_1−x_O_3_ do not occur; otherwise, Zr-depleted zones would be detected around the mulberry shaped structures. Hence, the maximum amount of Zr included into BaZr_x_Ti_1−x_O_3_ formed here is too small to cause reliably evaluable effects in the XRD patterns presented in Figure 1. The element maps of C and B (both not presented) also showed little contrast, whereas the distribution of Al (not presented) matched that of Si. The phase of dark contrast and dendritic morphology in the SEM micrographs should be enriched in light elements, i.e., B, Al, Si and possibly Na. Given the limited occurrence of these secondary phases in the overall volume of these samples, it is not surprising that they are basically not discernible in the XRD-patterns presented in Figure 1.

**Figure 5:**
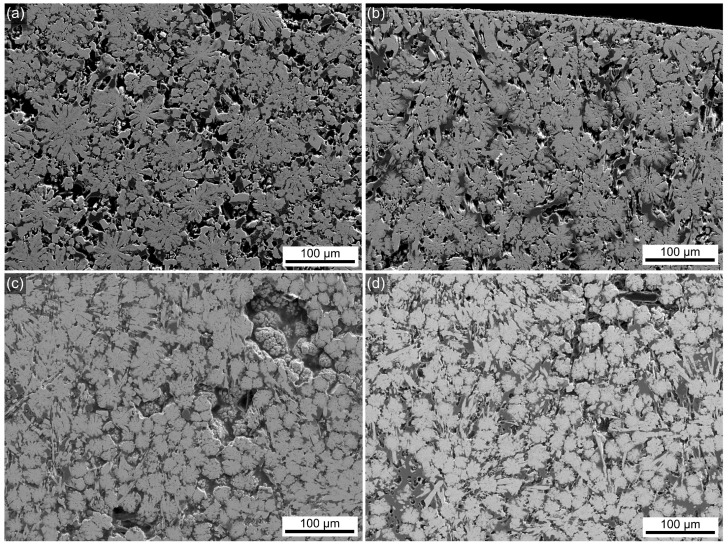
SEM micrographs of samples with (**a**) 0.5, (**b**) 1, (**c**) 2, and (**d**) 3 mol% ZrO_2_ heat-treated for 24 h at 680 °C.

Increasing the heat treatment temperature to 680 °C results in glass–ceramic samples which again contain the mulberry-shaped structures, but these are larger and more segmented as seen in Figure 5. These structures have collided with each other, indicating a more complete phase separation. Please note that phase-separated domains basically cannot show further growth once phase separation is completed [3]. This feature is typical both for the 0Zr crystallized samples and for the ZrO_2_-containing glass–ceramics 05Zr-3Zr.

**Figure 6:**
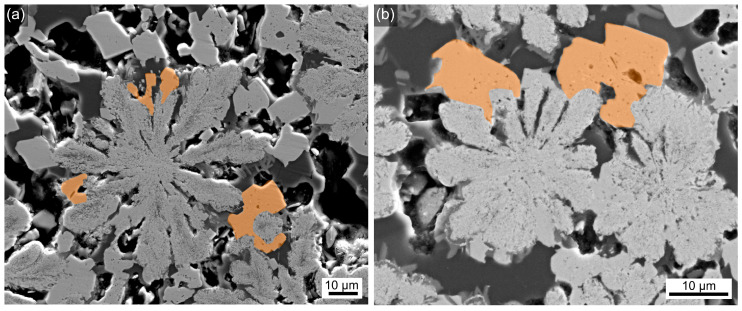
SEM micrographs of samples with (**a**) 0.5 or (**b**) 3 mol% ZrO_2_ heat-treated for 24 h at 680 °C. Selected fresnoite crystals in direct contact to the mulberry structures have been highlighted in orange.

A closer look at these microstructures reveals that some of the compact, blocky fresnoite grew directly onto the mulberry-shaped structures; two examples are presented in Figure 6. It should be noted that the presence of fresnoite in the samples annealed at 650 °C is confirmed by the XRD patterns shown in Figure 2. As both BaZr_x_Ti_1−x_O_3_ and fresnoite show a comparable attenuation cross-section (~1.5–2.0 cm^2^/g at the maximum intensity of the X-ray tube later used for XTM), they will show a similar contrast in XTM as they do in the SEM micrographs. Hence, the fresnoite crystals exemplarily highlighted in orange should affect the XTM signal, implying growth of the mulberry-shaped structures beyond their boundaries set by the initial phase separation. This would, however, be an artefact for lack of phase discrimination: any size increase is the combined effect of mulberry-shaped structures and compact fresnoite, but only the volume fraction of fresnoite actually increases for longer annealing times.

### 3.3. X-Ray Computed Tomography

**Figure 7:**
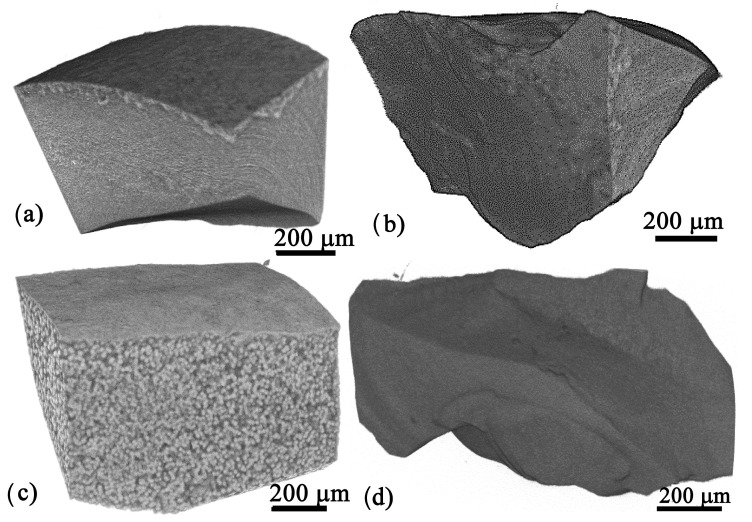
X-ray tomograms of samples with (**a**) 0.5 mol% and (**b**) 1 mol% ZrO_2_ annealed for 3 h at 590 °C; (**c**) 1 mol% and (**d**) 3 mol% ZrO_2_ heat-treated for 24 h at 590 °C.

**Figure 8:**
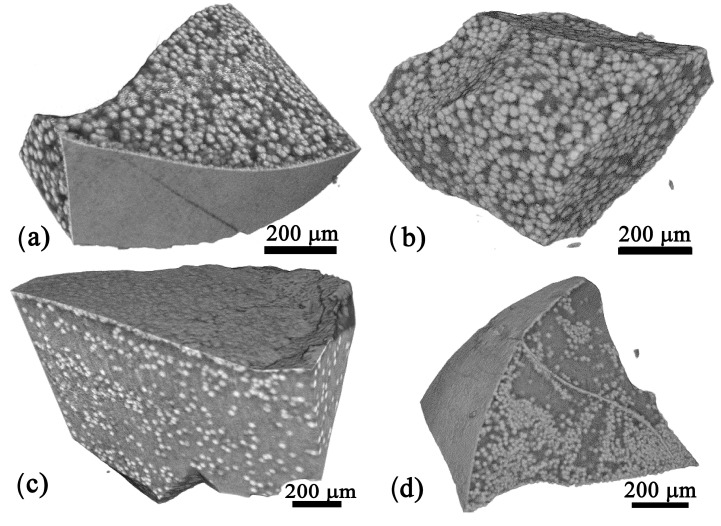
Tomograms of samples with (**a**) 0.5, (**b**) 1, (**c**) 2, and (**d**) 3 mol% ZrO_2_ heat-treated for 30 min at 680 °C.

**Figure 9:**
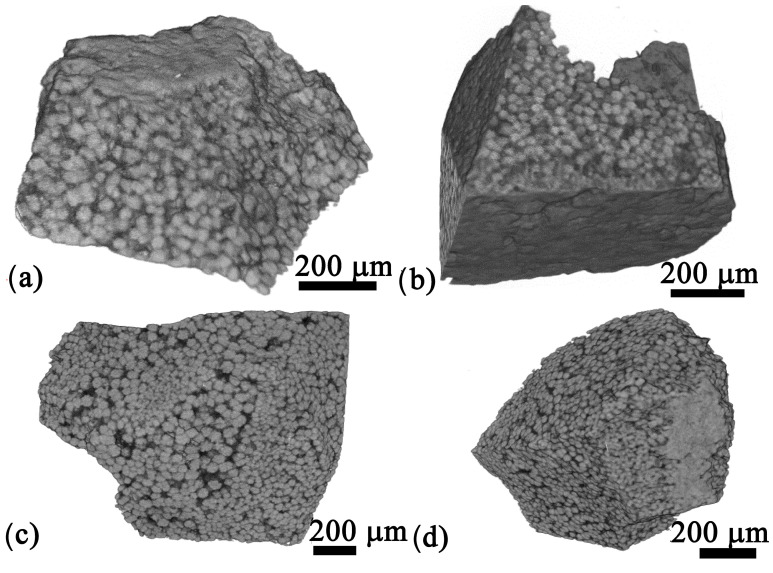
Tomograms of samples with (**a**) 0.5, (**b**) 1, (**c**) 2, and (**d**) 3 mol% ZrO_2_ heat-treated for 3 h at 680 °C.

As the average size, size distribution, and volume fraction of the dielectric phase(s) are important for the dielectric properties of the prepared glass–ceramics, the 05Zr-3Zr glass–ceramics with different thermal histories were analyzed using XTM. The results for the irregularly shaped pieces with different ZrO_2_ concentrations annealed at 590 °C for 24 h, i.e., when the fresnoite phase is hardly detectable in the XRD patterns, and at 680 °C for 30 min and 3 h, i.e., just before and after the fresnoite peaks become discernible in the XRD patterns, are shown in Figure 7, Figure 8 and Figure 9. The XTM images confirm the information from the SEM micrographs and show that all glass–ceramics obtained after heat treatment at 680 °C contain crystalline structures of bright contrast in large quantities, while most of the samples crystallized at 590 °C seem to be relatively homogeneous in the XTM analysis.

Samples prepared from the 05Zr and 1Zr glasses after annealing for 3 h at 590 °C appear rather homogeneous, with small changes discernible at the cast surface, as seen in Figure 7a,b. The sample 1Zr after heat treatment at 24 h at 590 °C shows easily visible bright precipitates in bulk and some changes at the as-cast surface, while the glass–ceramic obtained from 3Zr after thermal treatment at 590 °C for 24 h again appears comparably homogeneous in Figure 7d.

Figure 8a shows the sample prepared from the 05Zr glass and annealed for 30 min at 680 °C, containing spherical bright precipitates and some formations on the cast surface, which could be attributed to the surface crystallization. The 1Zr sample with the same thermal history only shows bright spherical particles, as the cast surface is not present here. The 2Zr sample shown in Figure 8c again features bright spherical precipitates both at the cast surface and in the bulk. The 3Zr sample is characterized by a heterogeneous distribution of the bright spherical precipitates in Figure 8d, which should be caused by chemical heterogeneity. Alternatively, it could also result from different sizes of bright precipitates, some of which may not be discernible via X-ray tomography due to its optical resolution of 4–5 µm for the device used.

The spherical precipitates in the X-ray tomograms of Figure 9 seem to be relatively homogeneously distributed and fill a relatively large volume fraction. The sizes reach the order of some 10 µm, keeping in mind that they contain both BaTiO_3_ and fresnoite, as outlined in Figure 6. It is characteristic for the samples prepared from the 05Zr, 1Zr, and 2Zr glasses that they all contain more or less uniformly distributed spherical precipitates in the sample volume, while the sample of 3Zr shows a smaller number of spherical precipitates.

### 3.4. Electrical Impedance Spectroscopy

The impedance spectra of glass–ceramic samples predominantly containing BaZr_x_Ti_1−x_O_3_ were recorded as Bode and Nyquist plots from 25 to 200 °C in the frequency range of 10 Hz–100 kHz. Fixed frequency measurements at 10, 50, 100, 500, 10^3^, 5 × 10^3^, 10^4^, 5 × 10^4^, 10^5^, and 5 × 10^5^ Hz were subsequently performed, assuming RC in parallel. The complex permittivity (ε) and the loss tangent (tanδ) were then calculated from the measured capacitance and resistance, taking the sample geometry into account. The real and imaginary part of the complex permittivity are evaluated using Equations (1) and (2). The loss tangent values were estimated using Equation (3). Equations (1)–(3) are as follows:

(1) $$\varepsilon^{\prime }\;=\;\frac{\mathrm{Cd}}{\varepsilon_{0}S}$$

(2) $$\varepsilon^{\prime \prime }\;=\;\frac{d}{{\omega \mathrm{RS}\varepsilon }_{0}}$$

(3) $$\tan\delta \;=\;\frac{\varepsilon^{\prime \prime }}{\varepsilon^{\prime }}$$

where C is the capacitance, ε_0_ is the vacuum permittivity, d is the separation between the plates of a capacitor, S is the area of the plates of a capacitor, R is the resistance, and ω is the angular frequency.

**Figure 10:**
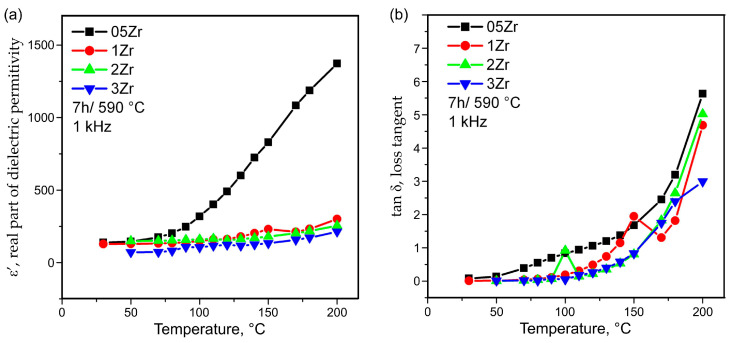
Dielectric constant (**a**) and loss tangent (**b**) at 1 kHz as a function of temperature for the glass–ceramic samples obtained from 05Zr-3Zr glasses after heat treatment for 7 h at 590 °C.

**Figure 11:**
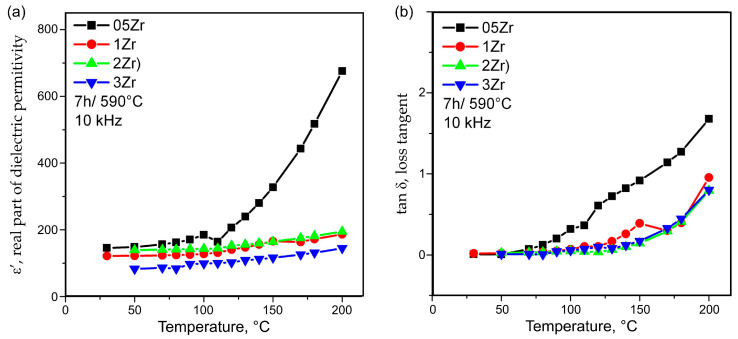
Dielectric constant (**a**) and loss tangent (**b**) at 10 kHz as a function of temperature for the glass–ceramic samples obtained from 05Zr-3Zr glasses after heat treatment for 7 h at 590 °C.

The results for the temperature dependence of the real part of the permittivity, ε′, and the loss tangent, tanδ, for frequencies of 1 and 10 kHz are given in Figure 10a,b and Figure 11a,b, respectively.

The glass–ceramics obtained after heat treatment at 680 °C were very brittle and cracked easily during annealing or the attempts to prepare plane parallel discs from them for the electrical measurements. Thus, it was not possible to perform impedance measurements on them until now.

## 4. Discussion

**Figure 12:**
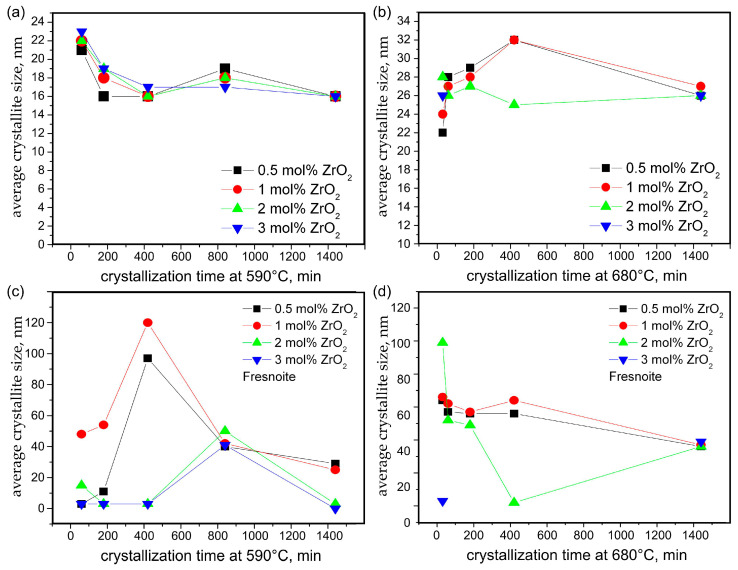
Average crystallite size from XRD line broadening as a function of the crystallization time after annealing at (**a**) 590 °C and (**b**) 680 °C for BaZr_x_Ti_1−x_O_3_ and (**c**) 590 °C and (**d**) 680 °C for Ba_2_TiSi_2_O_8_ in the case of glass–ceramics with different ZrO_2_ concentrations. Connecting lines are guides for the eyes.

The Scherrer equation [27] was used to estimate the average crystallite sizes for the BaZr_x_Ti_1−x_O_3_ crystals in the glass–ceramic samples with equal thermal history as a dependence on the ZrO_2_ concentration and the crystallization time. The determined average sizes for the BaZr_x_Ti_1−x_O_3_ and Ba_2_TiSi_2_O_8_ phases are presented in Figure 12 and in Table S1 in the Supplementary Material. These sizes vary slightly with the changing ZrO_2_ concentration and have a complicated trend of change for the lower annealing temperature of 590 °C. The average crystallite size of BaZr_x_Ti_1−x_O_3_ after annealing at 590 °C is practically independent from the ZrO_2_ concentration and initially decreases with increasing annealing time. For annealing times near 14 h, the crystallite size increases and then starts to decrease again. Nevertheless, all the crystallite sizes are in a relatively narrow range. As observed in similar BaTiO_3_-containing glass–ceramics [28] and reported for nanocrystalline BaTiO_3_ [29], the size of the crystallites and the change in the unit cell parameters for small crystallites depend on the kinetics of crystal growth. This tendency, however, is absent from the higher heat treatment temperature. The BaZr_x_Ti_1−x_O_3_ crystallite sizes increase with the increasing heat treatment time for the temperature of 680 °C in case of BaZr_x_Ti_1−x_O_3_; see Figure 12a,b. It is only at the highest annealing time of 24 h at 680 °C that the BaZr_x_Ti_1−x_O_3_ crystallites become smaller due to the growth competition at this higher temperature between the primary perovskite and the subsequently precipitated fresnoite crystals. Nevertheless, the respective diffraction domains of the BaZr_x_Ti_1−x_O_3_ crystallites remain relatively small and ≤ 35 nm, due to restricted growth possibilities resulting from the phase separation preceding the crystallization of this phase. The compact fresnoite crystals grow under different conditions, i.e., they initially start to grow at the as-cast surface and later also nucleate in the bulk. Thus, they are less constrained in their growth and are characterized by strongly varying sizes depending on the thermal history and to some extent on the zirconia concentration, as shown in Figure 12c,d and in Table S1 in the Supplementary Material. For the 05Zr- and 1Zr-type glass–ceramic samples, the crystallites of the fresnoite phase reach average sizes of ≥100 nm, where the Scherrer equation no longer gives reliable results. For the 2Zr and 3Zr glass–ceramics, the fresnoite crystals are smaller, as it could be surmised from the XTM results. Thus, it is not surprising that the average crystallite sizes of this phase are smaller for the 2Zr and 3Zr glass–ceramics as obtained from the Rietveld data refinement for both heat treatment temperatures.

The information presented so far already enables us to formulate a growth mechanism for these glass–ceramics: In the parent glass, a first phase separation into two liquids is observed. One of these liquids forms the mulberry-shaped structures which subsequently crystallize to contain the BaZr_x_Ti_1−x_O_3_ solid solution. The second liquid shows a strong surface crystallization of fresnoite and a crystallization of a very small fraction of another phase composed of comparably light elements. The crystallization of fresnoite in the bulk matrix occurs later on; a time gap between the surface and bulk crystallization of fresnoite in glass–ceramics is well-documented [30]. Although the structures of fresnoite and BaZr_x_Ti_1−x_O_3_ look similar in the presented micrographs, they are very different in detail, as fresnoite forms large, compact crystals at the µm scale, whereas the mulberry-shaped structures contain orientation domains of tiny crystals at the nm scale, as described in detail in Ref. [26] and observed in Figure 6.

XTM of the Zr-containing glass–ceramics enabled us to gather additional information on the average size of the crystallized volumes as a function of the annealing time and ZrO_2_ concentration; the results are stated in Table 2. This approach is superior to analyzing 2D cross-sections e.g., via SEM micrographs, as the internally connected mulberry-shaped structures can easily be cut apart and, thus, simulate a larger number and a smaller size of the structures than actually occur. Size evaluation must be performed keeping in mind that compact fresnoite grows onto the mulberry-shaped structures and increases their size, as outlined in Figure 6. In the 2Zr sample, a steep increase in the volume fraction of the crystalline phase from 17 to 61 vol% is observed when prolonging the crystallization time from 0.5 to 3 h at 680 °C. This is attributed to a factor of 3.6, while in all other samples, this factor was <1.6.

**Figure 13:**
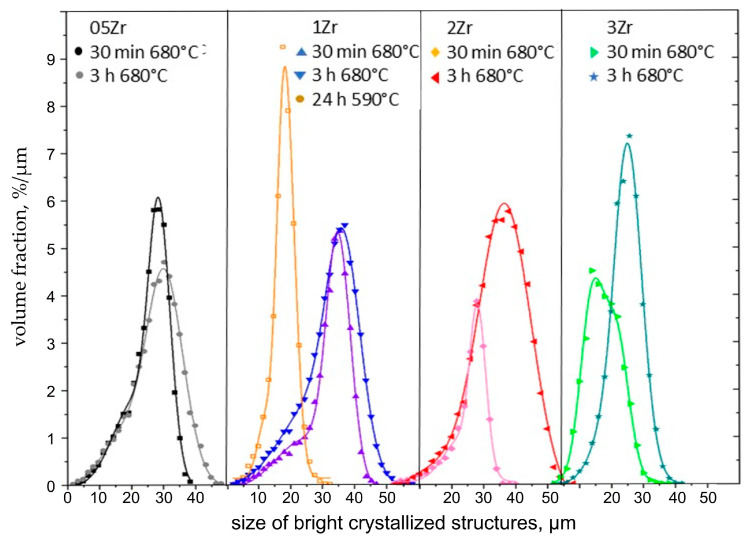
Size distributions of the crystallized structures composed of both BaZr_x_Ti_1−x_O_3_ and fresnoite determined from XTM as a function of the ZrO_2_ concentration and the thermal history.

The XTM results in Figure 8 and Figure 13 show that increasing ZrO_2_ concentrations lead to a decreasing size of the structures composed of both BaZr_x_Ti_1−x_O_3_ and fresnoite. This observation is explained by the results reported in the literature [22,23], which describe that replacing BaO with ZrO_2_ increases the network connectivity of a silicate glass. The coordination of Zr^4+^ ions in silicate and borosilicate glasses is known to be 6-fold compared to oxygen [31,32,33,34], and it has already been observed by other authors working with similar oxide compositions that despite its octahedral coordination, the addition of Zr to the composition results in increased network connectivity [33,34]. As the crystallite size of BaZr_x_Ti_1−x_O_3_ remains <35 nm, as shown in Figure 12, and BaTiO_3_ was not located outside of the phase-separated mulberry-shaped structures which cannot grow, the size increase in the crystallized structures in Table 2 should mainly be due to the growth of fresnoite. Not only do the fresnoite crystals increase in size themselves, but they can also fill the space amongst neighboring mulberry-shaped structures. This means that multiple structures can be “welded” together and form the much larger superstructures detected during XTM analysis, widening the possible sized beyond what is possible from normal crystal growth. In fact, the results in Figure 13 match the outlined process, as the samples heat-treated at 680 °C for different time periods are characterized by broader size distributions, and some of the curves may be deconvoluted into two or three separate curves, while the sample 1Zr annealed at 590 °C for 24 h is characterized by a single peak in Figure 13. The envelope curves from Figure 13 show the result from the Gauss fits of glass–ceramics, which have broader and non-symmetric peaks, but there is actually no theoretical reason for such a deconvolution of the curves.

Usually, the higher volume fraction and larger average crystallized structure size of a dielectric phase should result in higher dielectric constants and a better performance of the respective material. However, the increase in the crystalline volume fraction in the glass–ceramics prepared here is mainly achieved by the growth of fresnoite. Other authors working on the preparation of borosilicate glass–ceramics containing barium titanate or barium–strontium titanate have also reported the simultaneous occurrence of BaTiO_3_/Ba_1−z_Sr_z_TiO_3_ and Ba_2_TiSi_2_O_8_ as well as other crystalline phases [6,7,8,9], but no potential effect of the secondary crystalline phases is outlined in these works. Fresnoite has a much lower dielectric constant than BaTiO_3_ [30] and is known to be non-ferroelectric [35] and piezoelectric [30]. However, fresnoite manifests non-linear optical properties, i.e., photoluminescence in the blue spectral region and generation of a second harmonic [36,37] and it could be a new challenging task to study the controlled crystallization of two crystalline phase—one with high dielectric constant, i.e., BaZr_x_Ti_1−x_O_3_, and another one, Ba_2_TiSi_2_O_8_, with non-linear optical properties—in one and the same material with respect to opto-electronic applications.

The results from the investigation of the phase composition and the microstructure served as the basis for the interpretation of the data from Figure 10 and Figure 11, where the temperature dependence data of the dielectric constant and the dielectric losses of some of the prepared glass–ceramics are shown. The temperature dependence of the dielectric constant is characterized by the presence of one broad and not very high hump between about 90–160 °C, which is easily discernible for the 1Zr glass–ceramic sample and less visible for the 2Zr one. It is, however, very broad and vague for the 3Zr glass–ceramic and actually absent for the 05Zr type of glass–ceramics. Such a diffuse phase transition for the 1Zr and most probably for 2Zr and a vague one for 3Zr, as in Figure 10a and Figure 11a, can be attributed to a relaxor-type behavior [17,38,39,40,41,42,43]. Relaxor-type diffuse phase transitions are associated with the dispersion of the dielectric polarization over a broad temperature interval and the existence of relaxation effects over a wide temperature range which could have potential for practical applications. This type of phase transition could be due to compositional fluctuations, especially in cases of a deliberate addition of ions modifying the barium titanate crystals, or they might be caused by grain-boundary effects, as discussed in Ref. [42]. In the present samples, the relaxor phase transition, considering the temperature interval in which it occurs, can be attributed to a phase transition from tetragonal to cubic BaZr_x_Ti_1−x_O_3_ [44] and is similar to the temperature range observed by other authors preparing modified or pure BaTiO_3_ via the controlled crystallization of oxide glasses [4,5,6,7]. However, the results reported in [6,7,8,9] are for glass–ceramics with much larger grains than those shown here in the SEM micrographs. This difference in the grain sizes could be one possible explanation for the occurrence of the relaxor-type diffuse phase transition in the presented samples. However, the data from the XRD patterns reported in Figure 1 and Figure 2, as well as the EDXS results shown in Figure 4, suggest that compositional fluctuations and the incorporation of some of the Zr^4+^ ions into the BaTiO_3_ crystals might also be the reason for the observed relaxor-type transitions. It should be noted that the 05Zr glass–ceramic sample has the largest crystalline particles and the highest dielectric constant, but offers no hints as to the presence of some even vague phase transitions in the temperature range 90–160 °C, which cannot be explained by the currently available data on its dielectric properties. Otherwise, as expected, the dielectric constants decrease with the increasing frequency for one and the same temperature, and increase with increasing temperature, becoming very high for the 05Zr-type glass–ceramics and monotonously increasing for the 1Zr-3Zr glass–ceramics. Such behavior was also observed when preparing thin films of Zr-substituted barium titanate in [44], where the initial increase in the dielectric constant with the increasing Zr substitution was also observed, but then both the dielectric constant and the Curie temperature of the tetragonal- to cubic phase transition decreased for the reported Zr-substituted barium titanate. The monotonous increase in the dielectric constant in the 1Zr-3Zr glass–ceramics could also be associated with the glass network stabilizing role of the Zr^4+^ ions remaining in the amorphous matrix after the crystallization is initiated. The more rigid glass network should be associated with higher local viscosity and, thus, slower crystal growth. The dielectric losses follow the same trend with the increasing ZrO_2_ concentration. Namely, the glass–ceramic with the lowest ZrO_2_ concentration has the highest loss tangent for one and the same thermal history, while for the samples with zirconia concentration ≥ 1 mol% ZrO_2_, the dielectric losses remain practically the same for all the 1Zr-3Zr glass–ceramics.

## 5. Conclusions

Glasses were obtained for all ZrO_2_ concentrations in the compositional series 20.1 Na_2_O∙(23.1-y) BaO∙y ZrO_2_∙23 TiO_2_∙ 9.8 B_2_O_3_∙21 SiO_2_∙3 Al_2_O_3_ for y ≤ 3 mol% ZrO_2_. They phase separated into mulberry-shaped structures, which subsequently crystallized to contain BaZr_x_Ti_1−x_O_3_ with a crystallite size ≤ 35 nm, and a matrix in which fresnoite and another phase-containing light elements crystallized. Longer annealing times and higher temperatures led to an increasing content of fresnoite. Increasing ZrO_2_ concentrations suppressed the crystallization of fresnoite for lower heat treatment temperatures but had no significant impact at higher temperatures, e.g., 680 °C.

The growth of fresnoite can combine multiple phase-separated regions into much larger superstructures which are then detected as one feature by XTM. An increase in the ZrO_2_ concentration results in increased glass network connectivity and finer crystalline structures in the volume of the glass–ceramics.

The dielectric constants and loss tangents of the obtained materials have a complex dependence on the increasing zirconia concentration and exhibit a relaxor-type temperature dependence, which is attributed to the structural incorporation of the Zr^4+^ ions both in the amorphous matrix and their presence in the precipitated dielectric crystalline phase. These results reveal potential for electronic applications.

## Figures and Tables

**Table 1:** Glass transition, T_g_, and crystallization, T_c1_ and T_c2_, temperatures as a dependence on the ZrO_2_ concentration, according to [24].

Sample Name	T_g_, °C ±3 °C	T_c1_, °C ±3 °C	T_c2_, °C ±3 °C
05Zr	433	585	670
1Zr	443	585	683
2Zr	447	590	688
3Zr	450	590	683

**Table 2:** Fraction of the crystallized volume with a bright contrast in the glass–ceramics determined from the XTM tomograms depending on the thermal history and the ZrO_2_ concentration.

Sample Name	Thermal History, Time/Temperature	Average Bright Structure Size, µm	Bright Structure Volume Fraction, vol% (±15%)
05Zr	0.5 h/680 °C	26 ± 6	45 ± 7
05Zr	3 h/680 °C	28 ± 8	51 ± 8
1Zr	24 h/590 °C	18 ± 9	43 ± 6
1Zr	0.5 h/680 °C	31 ± 7	40 ± 6
1Zr	3 h/680 °C	32 ± 9	61 ± 9
2Zr	0.5 h/680 °C	17 ± 5	17 ± 3
2Zr	3 h/680 °C	35 ± 8	61 ± 9
3Zr	0.5 h/680 °C	17 ± 5	32 ± 5
3Zr	3 h/680 °C	25 ± 5	43 ± 6

## Data Availability

The original contributions presented in this study are included in the article/Supplementary Material. Further inquiries can be directed to the corresponding author.

## Supplementary Materials

The following supporting information can be downloaded at: https://www.mdpi.com/article/10.3390/ma18163783/s1, Table S1: Results from the Rietveld data refinement for the glass-ceramic samples with varying ZrO2 con-centrations.

## Abbreviations

The following abbreviations are used in this manuscript:

XRD	X-ray diffraction in the Bragg–Brentano configuration
SEM	Scanning electron microscopy
EDXS	Energy-dispersive X-ray spectroscopy
XTM	Microcomputed X-ray tomography
EBSD	Electron back-scatter diffraction
