# Supplementary material for: Barium Titanate-Based Glass–Ceramics Crystallized from Multicomponent Oxide Glasses: Phase Composition and Microstructure
Source: Materials (Basel). 2025 Aug 12;18(16):3783. doi: 10.3390/ma18163783 (PMC12387398; doi:10.3390/ma18163783)
Supplement: Supplementary file 1 [file materials-18-03783-s001.zip › materials-3776366-supplementary.pdf]

Supplementary material

Table S1. Results from the Rietveld data refinement for the glass-ceramic samples with varying ZrO_2 concentrations.

Sample name	Thermal history Time/temperature	Crystalline phase, wt%	$BaTiO_3$, wt%	$BaTiO_3$, Average crystallite size, nm	$Ba_2TiSi_2O_8$, wt%	$Ba_2TiSi_2O_8$, Average crystallite size, nm
05Zr	1h/590°C	1.53	96.3	21±0.2	3.7	3±0.4
1Zr	1h/590°C	1.66	99	22±0.3	1	48±0.4
2Zr	1h/590°C	1.44	98.1	22±0.4	1.9	15±1.5
3Zr	1h/590°C	1.36	97	23±0.4	3	≤ 3
05Zr	3h/590°C	5.44	95	16±0.2	5	11±1.3
1Zr	3h/590°C	7.04	97.6	18±0.4	2.4	54 ±0.2
2Zr	3h/590°C	6.32	96.3	19±0.3	3.7	≤ 3
3Zr	3h/590°C	5.38	94.1	19±0.4	5.9	≤ 3
05Zr	7h/590°C	9.9	96.8	16±0.2	3.2	97 ±2
1Zr	7h/590°C	8.31	98.5	16±0.5	1.5	120 ±4
2Zr	7h/590°C	7.42	96.5	16±0.3	3.5	≤ 3
3Zr	7h/590°C	10.15	96.6	17±0.4	3.4	≤ 3
05Zr	14h/590°C	11.22	93.7	18±0.4	6.3	40 ±2
1Zr	14h/590°C	13.04	93.2	18±0.4	6.8	42 ±1
2Zr	14h/590°C	10.57	92.9	18±0.3	7.1	50 ±2
3Zr	14h/590°C	9.72	90.4	17±0.5	9.6	41 ±1
05Zr	24h/590°C	10.5	89.4	16±0.2	10.6	29± 0.3
1Zr	24h/590°C	9.44	96.4	16 ±0.2	3.6	25±0.3
2Zr	24h/590°C	9.1	99.1	16 ±0.5	0.9	≤ 3
3Zr	24h/590°C	10.47	100	16±0.4	0	0
05Zr	30min/680°C	10.72	94.1	22±0.2	5.9	64±0.5
1Zr	30min/680°C	7.87	95.3	24±0.4	4.7	66 ±0.3
2Zr	30min/680°C	3.72	97.6	28±0.3	2.4	99±0.4
3Zr	30min/680°C	5.04	89.6	26±0.2	10.4	≤ 3
05Zr	1h/680°C	13.3	89.5	28±0.4	10.5	57 ±0.4
1Zr	1h/680°C	11.89	89.7	27±0.3	10.3	62 ±0.4
2Zr	1h/680°C	10.38	85.6	26±0.4	14.4	52 ±0.4
3Zr	1h/680°C	-	-	-	-	-
05Zr	3h/680°C	17.94	57.6	29±0.3	42.4	56 ±0.3
1Zr	3h/680°C	16.76	71.8	28±0.4	28.4	57 ±0.3
2Zr	3h/680°C	11.88	55.6	27±0.2	44.4	49 ±2
3Zr	3h/680°C	-	-	-	-	-
05Zr	7h/680°C	14.73	54.3	32±0.2	45.7	56 ±0.4
1Zr	7h/680°C	20.33	49	32±0.2	40.3 +10.7 SiO_2	64 ±0.4
2Zr	7h/680°C	13.51	55.3	25 ±0.4	44.7 +10.7 SiO_2	42 ±2
3Zr	7h/680°C	-	-	-	-	-
05Zr	24h/680°C	24.7	48.0	26 ± 0.1	49+3 ZrO_2	36 ±0.2
1Zr	24h/680°C	21.37	46.4	27 ±0.4	48.3 +0.6 TiO_2 (anatase)+4.8 ZrO_2	37 ±0.3
2Zr	24h/680°C	20.37	49.2	26±0.5	49+1.7ZrO_2	36±0.4
3Zr	24h/680°C	20.28	62	26 ±0.3	37.5+0.5ZrO	39 ± 0.5
